# Online tools for efficient paper writing

**DOI:** 10.1038/s41439-022-00195-9

**Published:** 2022-06-06

**Authors:** Yasunori Yamamoto, Toyofumi Fujiwara

**Affiliations:** Database Center for Life Science (DBCLS), Research Organization of Information and Systems (ROIS), 178-4-4 Wakashiba, Kashiwa, Chiba 277-0871 Japan

**Keywords:** Computational biology and bioinformatics, Medical research

## Abstract

For researchers, writing a paper is an essential task, and it is crucial for them to have an environment to facilitate the paper writing process. In addition, writing in English is more difficult for many non-native English speakers. The Database Center for Life Science (DBCLS) provides researchers in the life sciences with several text-mining related services, such as Allie and inMeXes, which were developed to facilitate paper writing. Allie is an abbreviation database that shows researchers expanded forms and several relevant data, such as the papers that contain the abbreviations and their corresponding expanded forms. Since a large amount of abbreviations are coined, remembering their meanings is difficult, even in one’s research field. Therefore, Allie helps one lookup abbreviations. inMeXes is an incremental search service for English phrases appearing in PubMed. Researchers can learn English phrases used in life science papers, such as the use of prepositions or widely used phrases that contain a specific word. Allie and inMeXes are updated monthly and yearly, respectively, to provide the latest information.

## Introduction

Many papers have been published in the life sciences due to the advancement of research instruments, such as next-generation sequencers. PubMed is a widely used biomedical bibliographic database containing more than 33 million entries. Moreover, on a daily basis, the entry count increases by more than three thousand on average (https://www.nlm.nih.gov/bsd/medline_pubmed_production_stats.html). Although reading a paper relevant to the researcher’s interest is indispensable, it is not easy to catch up with the latest findings^1^. Additionally, publishing a paper is a major task in research; therefore, writing a research paper efficiently is important.

As such, information technology can increase the efficiency of paper writing. The Database Center for Life Science (DBCLS) has provided several text-mining services to assist researchers in finding relevant information. These include Allie^2^, an abbreviation database, and inMeXes, an English phrase database.

First, Allie provides abbreviation-related information, such as the expanded forms, papers where abbreviations and their expanded forms appear, abbreviations regarding the main research field, and co-occurring abbreviations that appear in the same titles or abstracts. Researchers cannot always remember expanded forms or meanings because many abbreviations are used and created. Therefore, we developed Allie to help researchers easily search for the abbreviation definitions. Allie previously extracted pairs of an abbreviation and its expanded form from the entire PubMed database. Presently, more than 90,000 abbreviations appear more than 10 times. Additionally, Allie shows Japanese expressions of expanded forms for some that appear frequently.

Second, inMeXes provides an incremental search service for phrases used in PubMed. The results display any phrases exactly or partially matching an input string of the minimum length of three letters. The phrases are in descending order of appearance frequency, and therefore, users can learn commonly used phrases. Each phrase is an anchor text to link related information selected by a user, such as an online dictionary or a life science database. Indexed phrases are *n*-grams of the entire PubMed data (titles and abstracts). Here, *n* ranges from two to ten, and each *n*-gram appears at least ten times in PubMed. The main target users are researchers whose native languages are not English so that they can learn English phrases used in PubMed. Since PubMed is a collection of example phrases in life sciences, we believe that it is also useful for native English speakers. Additionally, inMeXes provides contextually similar words as an input string. For example, the words of *correlated*, *coincident*, and *compatible* are contextually similar to the word *associated* in PubMed. This feature is provided for researchers to search for alternative words that can be appropriate in the context when writing a paper.

The most noteworthy feature of these services is the frequent updates. Allie updates monthly, whereas inMeXes is updated annually. Additionally, Allie is freely downloadable as Resource Description Framework (RDF) data, which can be easily integrated with other RDF data, such as Medical Subject Headings (MeSH) RDF (https://id.nlm.nih.gov/mesh/) or UniProt (https://www.uniprot.org/format/uniprot_rdf/).

## Materials and methods

Both services employ the entire PubMed database, which is downloadable through the website of the National Library of Medicine (NLM) (https://www.nlm.nih.gov/databases/download/pubmed_medline.html). The PubMed data consist of multiple XML files, each of which has 30,000 bibliographic data entries, except for those with fractions. NLM releases a baseline set of PubMed data for download on a yearly basis. Additionally, NLM releases update files that include new, revised, and deleted data daily, and we can update the data derived from them. Each data entry has a title, abstract, authors, MeSH keywords, etc.

### Allie

Allie extracts pairs of an abbreviation and its expanded form from the PubMed titles and abstracts automatically^2^. In this paper, we briefly explain its procedure.

First, a tool called ALICE^3^ is used to extract them. ALICE is a rule-based information extraction (IE) system that analyzes a given text by applying a series of regular expressions one by one to find a pair. Its extraction performances are 95% recall and 97% precision on randomly selected titles and abstracts from the MEDLINE database. Since Allie’s performance depends on that of ALICE, pairs that ALICE cannot extract are not shown in the results of Allie. ALICE cannot extract a pair when it is expressed in the way ALICE does not assume. For example, for expansions divided by enumeration, in the string *topoisomerase I (topo I) or II (topo II)*, only *topoisomerase I (topo I)* can be identified.

Second, Allie aggregates the pair list obtained from ALICE. This is done using a tool called Carroll. Carroll employs a graph algorithm and groups identical pairs that are lexically varying by examining their concepts.

Additionally, a MeSH term and co-occurring abbreviations are extracted for each pair to help users select the most appropriate pair if there are multiple expansions for one abbreviation. In Allie, a MeSH term is used to indicate the major research area where a pair is used, and it is the most frequently annotated one in the papers that use the pair. Abbreviations co-occurring in the same paper act as a clue indicating if there are ones familiar to the users; thus, Allie groups them by paper. Allie also connects a pair and a paper that uses it to ensure that a user can learn when and where a pair is coined.

For the Japanese translations, we manually add them by referencing several dictionaries in life sciences and Japanese articles to gain reliability. For this reason, not all expanded forms have them. In addition, although the performance of the latest machine translation technology is high, we consider that provenance is important for translation, and we are not planning to use it for now.

### inMeXes

#### Incremental search

Since inMeXes shows a list of *n*-grams (*i.e*., parts of sentences) in PubMed, we need the sentences first. The downloadable PubMed XML data do not contain any sentence boundaries. Therefore, the first step is to split them into sentences. A sentence splitter called sptoolkit developed by Dr. Scott Piao is used for this task. The latest PubMed data have ~210 million sentences.

Next, *n*-grams of all sentences are generated and counted, where 2 ≤ *n* ≤ 10. This process employs a MapReduce-based tool (https://github.com/dbcls/inMeXes_Java) that the first author developed using Apache Hadoop. The number of *n*-grams is approximately 103 million.

The final step is to make these *n*-grams searchable. We use a compressed suffix array search tool called Sedue Flex and an open-source relational database management system called MySQL. Sedue Flex is a commercial and proprietary text search package developed and sold by Preferred Infrastructure. It consists of two tools, an indexer and a searcher. The former builds a custom-made index from given source text files. This index holds character positions in the files. The latter is a daemon program to accept a query text and return its start positions in the source files. Here, all *n*-grams are concatenated with a special character to delimit them, which are stored in a source file to be indexed.

**Fig. 1:**
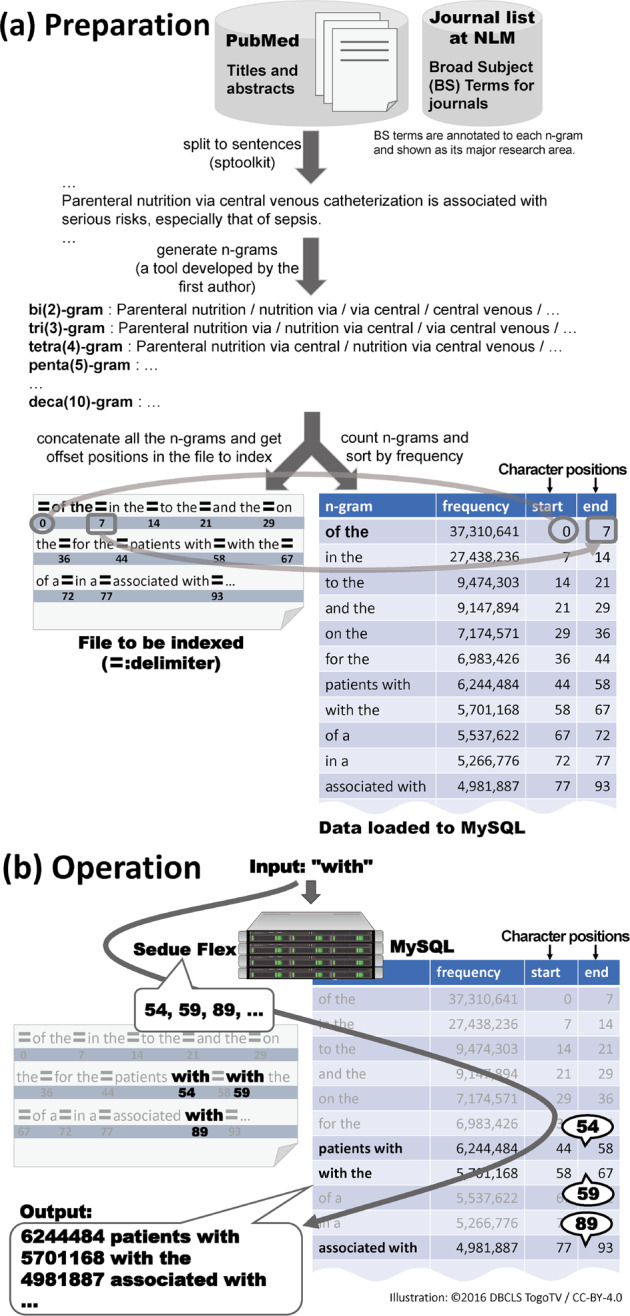
Preparation and operation of inMeXes. **a** A character index for Sedue Flex and a MySQL table to map character positions to *n*-grams along with appearance frequencies are built in the preparation phase. **b** Looking up positions from a given text and mapping from these positions to *n*-grams are operated in the operation phase.

MySQL stores *n*-grams and their start and end positions in the source file along with their appearance frequencies. Since Sedue Flex returns start positions of a matched text that is not necessarily an *n*-gram, MySQL maps these positions to *n*-grams that contain the text. That is, a position falls between the start and the end positions of an *n*-gram that includes the matched text. For example, a user gives a query *with* and Sedue Flex returns positions of its appearances in the source file. Then, MySQL returns the *n*-grams of *patients with*, *with*, *associated with*, etc., by comparing the stored positions to those given by Sedue Flex. All *n*-grams are concatenated in descending order of their appearance frequency in PubMed to ensure that the matching results shown to a user are ordered. Fig. 1 describes how to make an index (Preparation) and lookup phrases (Operation).

#### Contextually similar word search

inMeXes provides a service that shows a list of words appearing in contexts similar to a given word in PubMed. The list is ordered by cosine similarities between distributed representations of each one and the given word. These distributed representations are obtained using word2vec^4^, which is applied to the PubMed sentences for *n*-grams. The model is a continuous bag-of-words (CBOW) with a dimension of 100. For the PubMed 2021 baseline, the vocabulary size is 1,098,546, and the total words for training is 4,797,111,325.

## Results

These services have been provided for more than 10 years. Accompanied by an increase in the PubMed data, the number of entries continues to grow. Allie was launched in 2008, and there were fewer than 19 million PubMed entries; presently, there are more than 33 million. The latest version of Allie has 27,390,851 pairs and 4,556,427 unique ones. Additionally, the number of groups is 2,880,752. Allie provides a web-based search interface and application programming interfaces.

The latest version of inMeXes was built from the PubMed 2021 baseline. We have provided this service since 2009 and collect usage statistics from the access log from 2012 to 2021. The number of accesses used for analysis is almost four million (3,959,870). The analysis revealed that most users type in eight to nine letters within 1 s, indicating the readiness of inMeXes.

To introduce these services, DBCLS provides video tutorials, which are freely accessible. Those of Allie and inMeXes are https://togotv.dbcls.jp/20171117.html and https://togotv.dbcls.jp/20180328.html, respectively.

## Data Availability

Allie and inMeXes are available at the following URLs, respectively. https://allie.dbcls.jp/. https://docman.dbcls.jp/im/.
